# The Impact of COVID-19 Health Measures on Adults With Multiple Chemical Sensitivity: Cross-Sectional Study

**DOI:** 10.2196/48434

**Published:** 2024-07-17

**Authors:** Riina Bray, Yifan Wang, Nikolas Argiropoulos, Stephanie Robins, John Molot, Marie-Andrée Pigeon, Michel Gaudet, Pierre Auger, Emilie Bélanger, Rohini Peris

**Affiliations:** 1 Environmental Health Clinic Women’s College Hospital Toronto, ON Canada; 2 Association pour la santé environnementale du Québec - Environmental Health Association of Québec Saint Sauveur, QC Canada; 3 Faculty of Medicine University of Ottawa Ottawa, ON Canada

**Keywords:** COVID-19, multiple chemical sensitivity, Canada, accessibility, social isolation, physical environment, health care, air pollution, pollution, air quality, isolation, social network, social interaction, lived experience, sensitivity, environment, environmental

## Abstract

**Background:**

Multiple chemical sensitivity (MCS) develops in response to repeated small-level chemical exposures or a major exposure in a subset of people, who then experience symptoms that can range from mild to debilitating when exposed to chemicals. The arrival of the COVID-19 pandemic and the stringent health measures put in place may have increased the burden for those living with MCS, as it became more challenging to avoid chemicals that trigger their condition.

**Objective:**

This study aimed to better understand the lived experience of Canadians living with MCS during the first year of the COVID-19 pandemic.

**Methods:**

An online questionnaire was created to ask participants to compare daily living during the pandemic to before March 11, 2020. Data were collected in January and February 2021. Three areas were investigated: (1) environmental exposures to chemical triggers from ambient air (pollution from industry, farming, and traffic) and indoor air (the smell of cleaning products, cooking odors, and smoke); (2) access to, and satisfaction with, health care visits; and (3) how people experiencing MCS rated contact with their social network.

**Results:**

In all, 119 Canadians who had lived with MCS for more than a year completed the questionnaire. The participant sample was mostly female (86.6%, n=103) and highly educated, with 57.1% (n=68) having a university degree. Slightly more than half (57.1%, n=68) were older than 55 years. McNemar chi-square and Wilcoxon signed rank tests were used to evaluate if there were statistically significant changes before (“prepandemic period”) and after (“postpandemic period”) March 11, 2020. Perceived exposure to pollution from a highway or a road was significantly decreased from the prepandemic to postpandemic period (*z*=–3.347; *P*<.001). Analysis of industry or power plants also suggested a significant decrease in the perceived exposure from the prepandemic to postpandemic period (*z*=–2.152; *P*=.04). Participants reported an increase in exposure to odors from disinfectants or sanitizers that entered their living environment (*P*<.001). There was a significant decrease between prepandemic and postpandemic levels of satisfaction when attending in-person meetings with a physician (*z*=–2.048; *P*=.04), yet there were no significant differences between prepandemic and postpandemic levels of satisfaction for online or telephone meetings with a physician. Although people with MCS experienced increased social isolation (*P*<.001), they also reported an increase in understanding from family (*P*=.03) and a decrease in stigma for wearing personal protective equipment (*P*<.001).

**Conclusions:**

During the first year of the COVID-19 pandemic, people with MCS were impacted by inaccessibility, loss of social support, and barriers to accessing health care. This study highlights unique challenges and possible benefits associated with the COVID-19 pandemic public health measures for individuals living with MCS. These findings can guide decision makers to improve policies on accessibility through appropriate accommodation measures.

## Introduction

Multiple chemical sensitivity (MCS), also known as toxicant-induced loss of tolerance, has been described as a chronic condition that develops in response to repeated small-level chemical exposures or a major exposure in a subset of people [1-3]. After an “initiation” stage, the person reacts strongly to subsequent chemical exposures and may experience symptoms such as headache and difficulty concentrating, shortness of breath, nausea, stomach pain, skin irritation, nutritional deficiencies, and overall fatigue [4-7]. The pathophysiological mechanisms underlying the condition are still being researched and described in the scientific literature [4,5,8]. As there is no known diagnostic marker for MCS, the condition is diagnosed based on patient history and symptom reports alone [9].

The Canadian Community Health Survey of 2020 reports that over 1 million Canadians have a diagnosis of MCS, a diagnosis seen to predominantly affect women (72%), with approximately half of those diagnosed being older than 55 years of age [10]. From a public health perspective, MCS is a recognized disability [11] that can cause challenges for everyday living. The arrival of the COVID-19 pandemic may have increased the burden for those living with MCS, as avoiding chemical cleaners became nearly impossible [12]. “Pandemic products” such as cleaners and disinfectants [13] are now widely used and accepted by the public as a necessary part of infection prevention and control [14]. While the COVID-19 transmission rates have been improved by many of these measures, the detrimental effects of cleaners and disinfectants on health have been reported. Poison control centers in Canada and the United States have reported an increase in the total number of phone calls related to accidental exposures to toxic levels of cleaners since the beginning of the pandemic [15,16]. There has also been a decrease in indoor air quality as particulate matter exposure increased during the COVID-19 pandemic due to the increased frequency of cleaning and household activities [17]. To avoid products that may trigger symptoms, people experiencing MCS have reported limiting contact with others and avoiding going into their workplace, seeking health care [18], or shopping for groceries [19]. In the context of the COVID-19 pandemic, both the infection prevention and control measures and the virus represent a threat to the health of people experiencing MCS.

Accessing health care has become increasingly difficult with the emergence of COVID-19. Some populations were adversely affected by the reallocation of health care resources during the first few waves of the pandemic, including those with life-threatening conditions such as cancer [20] and heart disease [21], who received delayed diagnoses and care. For people with MCS, obtaining medical attention may have been challenging as their care may be viewed as a low priority. Despite this, these individuals live with comorbidities such as asthma, chronic obstructive pulmonary disease, and diabetes, which may increase their risk of developing severe COVID-19 symptoms [22,23]. For these same reasons, patients with MCS may have avoided vaccination clinics or urgent care facilities; according to the Centers for Disease Control and Prevention (CDC), 1 in 4 adults in the United States avoided medical visits during the first wave of the COVID-19 pandemic due to fear of infection, with a higher proportion among those with disabilities [24]. To our knowledge, how people experiencing MCS accessed medical care during the pandemic remains unknown.

The existing scientific literature describes aspects of the health, social, and economic functioning of people experiencing MCS [25-29]. A recurrent theme that emerges is the invisible and complex nature of chemical sensitivities, which makes it difficult to explain to friends or colleagues and is often contested by health care providers [30]. As a result, people experiencing MCS can be reticent to ask for accommodations for their condition as their symptoms may be trivialized or discounted entirely. According to the Accessible Canada Act of 2019, for people with impairments of any kind (eg, physical, cognitive, or sensory perception), the inability to participate fully and equally in society is considered a barrier to accessibility [31]. For people experiencing MCS, this barrier is most often felt when they must explain and request that the built environment be as nontoxic and scent-free as possible. Thus, the stigma of this condition, along with the burden involved in planning outings to safe shared spaces, can result in social isolation. In this light, the pandemic may have affected the lived experiences of people experiencing MCS in how they experienced shared living spaces, accessed health care, and maintained social connections.

The aim of this study is to better understand the lived experience of people experiencing MCS during the first year of the COVID-19 pandemic. Specifically, three domains are investigated in a cohort of people experiencing MCS: (1) environmental exposures to chemical triggers from ambient and indoor air (ie, how the living environment is perceived); (2) access to, and satisfaction with health care (eg, what concerns people experiencing MCS had with the physical space of medical facilities or staff); and (3) how people experiencing MCS rated contact with their social network, including issues of isolation and requests for accommodation for their MCS.

## Methods

### Study Design

A cross-sectional study design with a retrospective component was used to measure the impact of the COVID-19 pandemic before and after March 11, 2020, when the World Health Organization (WHO) officially declared COVID-19 a pandemic [32].

### Ethical Considerations

This study was approved by the Women’s College Hospital Research Ethics Board (study # 2020-0157-E). Participants were treated in accordance with the Declaration of Helsinki. All participants provided informed consent electronically. To maintain confidentiality, participant data were deidentified (identified on all databases with an ID number only). Participants were not compensated for their time.

### Recruitment and Procedures

Participants were a convenience sample of Canadian residents. To be eligible, they had to be older than 18 years of age; proficient in either English or French; and have experienced MCS symptoms for at least 1 year prior to March 11, 2020. Participant recruitment and data collection occurred between January 19, 2021, and February 12, 2021. Participants were recruited via 2 websites (Association pour la santé environnementale du Québec—Environmental Health Association of Québec [ASEQ-EHAQ] and Women’s College Hospital), on social media, and via email through the ASEQ-EHAQ mailing list. A link was embedded in the post or email that connected directly to the screening questionnaire. Participants could also visit the ASEQ-EHAQ website for further information and could access the screening questionnaire by clicking on the MCS or COVID-19 project page.

An initial online screening questionnaire determined eligibility, after which participants were either deemed ineligible and thanked for their interest or deemed eligible and provided with access to the consent form. Upon consenting to the study terms, participants accessed the questionnaire on the Qualtrics XM platform (Qualtrics, LLC), a secure web-based data collection survey tool. If a participant had any difficulty filling out the survey online, the participant contacted ASEQ-EHAQ by telephone to give verbal consent and the survey was administered over the phone. Informed consent was obtained from all participants. All study materials were available in both English and French.

A sample of 373 potential participants was screened. Of these, 254 (68.1%) did not meet inclusion criteria, while 119 (31.9%) did and agreed to complete the questionnaire.

### Questionnaire

The questionnaire was created in house by specialist physicians, expert scientists, and people with lived experience of MCS. To ensure content validity, the questionnaire was reviewed with input by people experiencing MCS and a panel of clinicians prior to the beginning of the study. Participants answered a total of 81 questions, of which 21 were retained for the purpose of this study.

The questionnaire asked participants to consider their lives from before the pandemic (“prepandemic period” being defined as before March 11, 2020) and at the time of the survey (“postpandemic period” being defined as after March 11, 2020) with a data collection window of January 19 to February 12, 2021. Participants answered questions regarding their sex, age, income, education, MCS symptomatology, and the time between symptom onset and diagnosis. To assess the impact of COVID-19 public health measures, first, participants were asked to indicate if they were exposed (yes, no, or unsure) to chemical triggers from their living and the outdoor environment (ie, in indoor and outdoor air). Living environment items included home cleaning products; cooking odors and smoke; and outdoor items included pollution from industry, farming, and traffic. Next, participants responded (yes or no) if they were able to access a family doctor or a doctor who treats MCS, both in-person and online health care visits. Satisfaction with visits was rated on a 5-point Likert scale from very dissatisfied to very satisfied. Possible barriers to attending any health care professional visits were assessed by asking if participants would be concerned (yes or no) with indoor air quality issues (renovations, disinfectant use, or scents from the building cleaning products), the risk of infection (social distancing, exposure to flu or colds), and being in contact with chemicals in new masks. Participants were also asked if they had requested accommodation for their condition (yes or no) and if this request had been met. Changes in social networking were assessed by asking participants to rate how often, on a 5-point Likert scale, they experienced social isolation, participation in the community, stigma from wearing a mask, support from others, or meetings with friends and family. Finally, 1 question assessed how difficult it was (difficult, moderate, or easy) to obtain help with tasks such as shopping or home maintenance. The questionnaire and response values can be found in Multimedia Appendix 1.

### Data Analysis

The data were downloaded from Qualtrics, deidentified, and imported into statistical software. Data were analyzed using SPSS Statistics (version 27; IBM Corp) predictive analytics software. An exact significance criterion of .05 (2-tailed) was adopted for all statistical tests. Due to the nature of the scoring, and the repeated measures design, repeated measures nonparametric analyses were conducted. Outliers were thus retained. Less than 10% of the values were missing, so no imputation was conducted. Descriptive statistics are presented as mean with SD, median with IQR, and counts with percentages. Statistical significance was assessed using the McNemar chi-square test for binomial distributions and Wilcoxon signed rank test for differences between responses before and after March 11, 2020.

## Results

### Demographics

Respondents’ (N=119) demographic characteristics are presented in Table 1.

**Table 1:** Demographic characteristics of people experiencing MCS^a^ (N=119).

Variables			Values
**Sex** **, n** **(%)**			
	Female	103 (86.6)	
	Male	16 (13.4)	
**Age group (years), n (%)**			
	25-34	9 (7.6)	
	35-44	23 (19.3)	
	45-54	19 (16)	
	55-64	43 (36.1)	
	64-74	19 (16)	
	≥75	6 (5)	
**Current work status, n (%)**			
	Employed	46 (38.7)	
	Unemployed	34 (28.6)	
	Student or retired	39 (32.7)	
**Household income^b^ (CAD $), n (%)**			
	<10,000	2 (1.9)	
	10,000 to 20,000	30 (28.6)	
	20,000 to 30,000	6 (5.7)	
	30,000 to 40,000	11 (10.5)	
	40,000 to 50,000	11 (10.5)	
	50,000 to 60,000	10 (9.5)	
	60,000 to 70,000	5 (4.8)	
	70,000 to 80,000	9 (8.5)	
	>80,000	21 (20)	
	Prefer not to answer	14 (11.8)	
**Education, n (%)**			
	Secondary school	9 (7.6)	
	College	40 (33.6)	
	University	68 (57.1)	
	Prefer not to answer	2 (1.7)	
Time since onset of symptoms (years), mean (SD)			20.22 (12.67)
Time period between onset and diagnosis (n=92; years), mean (SD)			8.39 (9.55)

^a^MCS: multiple chemical sensitivity.

^b^All dollars are listed in Canadian currency. The conversion rate for CAD $1 to US $ ranged from 0.78 to 0.79 cents between January 19 and February 12, 2021 (Bank of Canada currency converter).

### Exposure to Triggers From the Living Environment

The dichotomous (yes or no) prepandemic and postpandemic change in self-reported exposure to odors or other triggers was assessed using the McNemar chi-square test (Table 2). Of the 9 items participants were asked to assess, there was a statistically significant change (*P*<.001) in exposure to disinfectants or sanitizers; no others were significantly different.

**Table 2:** Changes in exposure to triggers from the living environment (indoor air quality).

Triggers and prepandemic levels^a^		Postpandemic levels^b^, n			*P* value^c,d^	
		No	Yes			
**Cleaning products**						>.99^c^
	No	37	10			
	Yes	10	56			
**Cooking odors**						.29^c^
	No	46	6			
	Yes	2	59			
**Disinfectants or sanitizers**						<.001^d^
	No	40	24			
	Yes	5	46			
**Scents**						>.99^c^
	No	32	5			
	Yes	6	71			
**Home renovation**						.08^c^
	No	63	4			
	Yes	12	27			
**Incense**						.23^c^
	No	79	3			
	Yes	8	18			
**Laundry products**						.18^c^
	No	24	2			
	Yes	7	82			
**Second- or thirdhand tobacco smoke**						.30^c^
	No	57	5			
	Yes	10	40			
**Second- or thirdhand marijuana smoke**						>.99^c^
	No	70	4			
	Yes	4	31			

^a^Before March 11, 2020.

^b^After March 11, 2020.

^c^Exact significance (2-tailed).

^d^Asymptotic significance (2-tailed).

### Exposure to Triggers From the Greater Environment (Ambient Air Quality)

Participants rated their perceived exposure to air pollutants from farming, industry or power plants, highways, or roads, and residential or commercial smoke. Differences were assessed using a Wilcoxon signed rank test. There were no significant differences when comparing prepandemic and postpandemic air pollution exposure ranks due to farming and residential or commercial smoke; however, perceived exposure to pollution from a highway or a road was significantly decreased from the prepandemic (median 2, IQR 0-2) to postpandemic period (median 0, IQR 0-2; *z*=–3.347; *P*<.001). Analysis of industry or power plants suggests a significant decrease in the perceived exposure from the prepandemic (median 0, IQR 0-1.75) to postpandemic period (median 0, IQR 0-0; *z*=–2.152; *P*=.04).

### Health Care

#### Health Care Accessibility

Participants’ ability to access a doctor, as well as items of concern regarding visiting medical facilities, are tabulated in Table 3. A McNemar chi-square test revealed that participants endorsed their access to a family doctor was statistically different (*P*<.001) after March 11, 2020. Similarly, analysis of discordant pairs suggests a statistically significant difference in access to a family doctor to address one’s MCS condition before and after March 11, 2020 (*P*=.03). Regarding access to a medical facility, there was a statistically significant difference in exposure to chemicals in new masks (*P*<.001), exposure to unscented disinfectants or sanitizers (*P=*.004), and exposure to scents in the building or premises (*P*=.006). Table 3 shows the complete results.

**Table 3:** Health care provider and facility accessibility before and after March 11, 2020.

Accessibility and prepandemic levels^a^			Postpandemic levels^b^, n									*P* value^c,d^
			No		Yes		Unselected		Selected			
**Access to a family doctor**												<.001^d^
	No	15		1		—^e^		—				
	Yes	16		87		—		—				
**Access to a family doctor who addresses MCS^f,g^**												.03^c^
	No	49		0		—		—				
	Yes	6		32		—		—				
**Air pollution exposure**												<.001^d^
	Unselected	—		—		70		0				
	Selected	—		—		12		37				
**Cleaning products used in the building or premises**												.002^c^
	Unselected	—		—		29		1				
	Selected	—		—		13		76				
**Distance to travel**												.003^c^
	Unselected	—		—		72		1				
	Selected	—		—		12		34				
**Exposures to chemicals in new masks**												<.001^d^
	Unselected	—		—		68		29				
	Selected	—		—		4		18				
**Exposures to unscented disinfectants or sanitizers**												.004^c^
	Unselected	—		—		66		16				
	Selected	—		—		3		34				
**Outbreak of flu or colds**												.34^c^
	Unselected	—		—		102		3				
	Selected	—		—		7		7				
**Physical distancing or wearing of masks or gloves inadequate**												<.001^d^
	Unselected	—		—		87		19				
	Selected	—		—		3		10				
**Exposures to scented disinfectants or sanitizers**												.61^c^
	Unselected	—		—		29		6				
	Selected	—		—		9		75				
**Exposures to scents in the building or premises**												.006^c^
	Unselected	—		—		24		1				
	Selected	—		—		11		83				
**Exposures to scents worn by health care workers or support staff**												.39^c^
	Unselected	—		—		36		4				
	Selected	—		—		8		71				
**Mold or water damage exposure in the building or premises**												.63^c^
	Unselected	—		—		96		3				
	Selected	—		—		1		19				
**Recent construction or renovations**												<.001^d^
	Unselected	—		—		87		0				
	Selected	—		—		11		21				
**Second- or thirdhand smoke exposures**												>.99^c^
	Unselected	—		—		82		6				
	Selected	—		—		5		26				

^a^Before March 11, 2020.

^b^After March 11, 2020.

^c^Exact significance (2-tailed).

^d^Asymptotic significance (2-tailed)

^e^Not available.

^f^MCS: multiple chemical sensitivity.

^g^Question only displayed if participants endorsed having access to a family doctor in the prepandemic and postpandemic period (n=87).

#### Satisfaction With Health Care

There was a significant decrease between the prepandemic (median 2, IQR 1-3) and postpandemic periods (median 2, IQR 0-3) in the levels of satisfaction when attending in-person meetings with a physician (*z*=–2.048; *P*=.04). There were no significant differences between prepandemic and postpandemic levels of satisfaction when attending online or telephone meetings with a physician.

#### Requests for Accommodation

A McNemar chi-square test was conducted to determine the relative change in people experiencing MCS requesting accommodation for their condition (see Table 4). The results suggest a statistically significant difference between requests before and after March 11, 2020 (*P*<.001). Overall, the majority (101/119, 84.9%) of people experiencing MCS requested accommodation for their MCS prior to March 11, 2020, whereas during the COVID-19 pandemic, requests for accommodation decreased to 58% (69/119).

When asked if, overall, their requests were reasonably accommodated across these categories, results show there were no significant differences (*P*>.99) between prepandemic and postpandemic levels of the request being met. This suggests that people experiencing MCS received the same level of accommodation when the request was made.

**Table 4:** Accommodation: requests and items of concern (reason the request was made for people experiencing MCS^a^; N=119).

Requested accommodation			Prepandemic levels^b^, n (%)		Postpandemic levels^c^, n (%)
**Reason the request was made**					
	Use of scent	86 (85.1)		49 (71)	
	Use of scented laundry products which enter the living space	61 (60)		33 (47.8)	
	Fumes from cleaning products used in common areas	54 (53.5)		28 (40.6)	
**Participants asked for**					
	Scent-free policies	82 (69)		37 (31.4)	
	Use of scent-free and least-toxic cleaning products	79 (66.4)		45 (37.8)	
	Supply of scent-free and least-toxic soaps, disinfectants, or sanitizers	69 (58)		37 (31.4)	

^a^MCS: multiple chemical sensitivity.

^b^Before March 11, 2020.

^c^After March 11, 2020.

#### Wilcoxon Signed Rank Test Analysis of Social Interactions

Participants were asked to rate their experience of social isolation, stigma, support, and obtaining assistance both before and after the pandemic began. Table 5 shows that 10 of 13 categories report significant differences.

**Table 5:** Self-reported changes to social networking by people experiencing multiple chemical sensitivity pre-post March 11, 2020.

Self-reported changes			Prepandemic scores, median (IQR)		Postpandemic scores, median (IQR)		*z* score	*P* value^a,b^
**Social isolation**								
	Feeling socially isolated	2 (1-3)		3 (2-4)		–6.020		<.001^b^
	Participation in the local community	1 (0-3)		0 (0-1)		–4.907		<.001^b^
	Stigma wearing masks, gloves, or other protective equipment	2 (0-4)		0 (0-1)		–5.683		<.001^b^
	Support and understanding from family	2 (1-3)		3 (1-3)		–2.265		.03^a^
	Support and understanding from friends	2 (1-3)		2 (1-3)		–0.481		.66^a^
	In-person meeting with friends	2 (1-3)		1 (0-1)		–7.633		<.001^b^
	In-person meeting with family	2 (1-3)		1 (0-1)		–7.288		<.001^b^
	Online relationships or meetings	1 (0-2)		2 (1-3)		–7.156		<.001^b^
**Level of difficulty of finding scent-free workers for the following task**								
	Shopping for groceries	2 (2-3)		2 (2-3)		0.584		.80^a^
	Driving to appointments or for (nongrocery) shopping	3 (2-3)		3 (2-3)		–2.310		.002^a^
	Home repairs or maintenance	2 (1-2)		2 (2-3)		–2.814		<.001^b^
	Home support	3 (2-3)		3 (2-3)		–0.333		.27^a^
	House cleaning	3 (2-3)		3 (2-3)		–1.265		.001^b^

^a^Exact significance (2-tailed).

^b^Asymptotic significance (2-tailed).

## Discussion

### Principal Findings

This study assessed how a cohort of Canadians with MCS experienced changes in their living environment, access to and experience of health care, and interactions in their social network before and after the COVID-19 pandemic was declared on March 11, 2020. Results indicate significant changes in all 3 areas and provide insight into how people with MCS experienced the effects of the COVID-19 pandemic in their daily lives.

The 119 participants in this study were predominantly female (n=103, 86.6%) and highly educated, with 57.1% (n=68) having a university degree, 33.6% (n=40) having completed college, and 7.6% (n=9) having completed secondary school. Slightly more than half (n=68, 57.1%) were older than 55 years of age, in line with Canadian statistics. The mean duration of MCS symptoms was 20.2 (SD 12.67) years, and the average time it took to receive a diagnosis of MCS was 8.4 (SD 9.55) years, suggesting that MCS began in early adulthood, but participants only received a diagnosis almost a decade later. For comparison, the mean time to diagnosis for fibromyalgia, a chronic condition that co-occurs frequently with MCS, is approximately 6.2 years [33].

### Principal Results

#### Indoor and Outdoor Air Quality—The Living Environment

Within days of the declaration of the pandemic, provinces across Canada implemented national public health measures to reduce the risk of viral transmission [34]. Isolation was mandated, which resulted in the closing of daycares, schools, and nonessential businesses, as well as limiting travel between regions; in Québec, an early evening curfew was imposed. Family members spent an increased amount of time indoors, posing a risk of contracting COVID-19 from another family member, but also risking exposure to poor indoor air quality [35]. Indoor air quality is a major contributor to human health and is significantly affected by human activities such as smoking, vaping, cooking, using appliances, heating and cleaning [36]. Participants in this study reported increased exposures to odors from disinfectants that entered their living environment, which may reflect the increased human density and duration of activities within nearby homes and multifamily dwellings, as well as the intensified cleaning that was carried out using products strong enough to inactivate COVID-19.

The risk to health from ambient and indoor air pollution has come into sharp focus with the COVID-19 pandemic [37-39]. Here, we report the added concern by participants of repeated exposure to odors from disinfectants that may trigger symptoms and may lead to an increase in sensitivity to chemicals, akin to the dose-response relationship between exposure to disinfectants and increased risk of asthma in adults and children [40]. Furthermore, fragranced products can emit volatile organic compounds associated with harmful effects on the neurological, gastrointestinal, respiratory, and immune systems [13]. As not all active ingredients are disclosed on product labels there is an additional risk to users’ health, especially when they are used without necessary caution [41]. This reinforces the importance of accurate labeling for fragranced, unscented (which may contain masking agents), and fragrance-free disinfectants, with appropriate symbols for toxicity, clear indications of optimal use, and warnings about the risk of emissions from mixing products together [42].

In Europe, a decrease in ambient air pollution observed during lockdown periods proved beneficial to overall health [43]. Ambient air analyses from ground-level urban Canadian cities revealed significant reductions in black carbon, nitrogen dioxide, and particulate matter under 2.5 microns (PM_2.5_) with an increase in ozone levels during the first phases of the pandemic [44]—in line with multiple reports from countries worldwide [45]. Consistent with this, we found a decrease in perceived outdoor pollution exposure from highways or roads and industry, likely due to the slowdown of economic activities. This reduction may have mitigated the effects of other odors perceived by people experiencing MCS in their homes, as outdoor air quality has a direct relationship with indoor air quality, which in turn has implications for human health [46,47].

#### Health Care

As COVID-19–related illnesses and mortality increased, health care resources were reallocated to its treatment and prevention, resulting in reduced access to nonurgent care, diagnostics, surgeries, and other health services, even in countries with strong health care systems [48]. Participants reported a reduction in access to a general practitioner for health care, as well as the ability to reach a physician to address their MCS. Results also demonstrate that people experiencing MCS were more concerned that in-person clinic visits might expose them to chemicals in new masks, hand sanitizers, and disinfectants. There is evidence that increased mask and disinfectant use worsens symptoms in individuals with chronic conditions such as migraine headaches or asthma [49,50]. Furthermore, a barrier to health care for people experiencing MCS is that the clinical and therapeutic management of MCS is complex and multifaceted [51], imposing an additional burden on interprofessional collaboration within an already strained health care system. People experiencing MCS may have postponed or abandoned attempts to access routine health care for these reasons, which put them at greater risk for delayed diagnosis and treatment of illness. The WHO has stressed the importance of providing accessible environments for people with disabilities [52], yet barriers intensified for those with disabilities during the pandemic, especially in terms of health care [53]. Despite this, participants did not report a change in satisfaction with telephone or video appointments. Telehealth may be an important avenue in the future of MCS care, obviating the need for in-person visits (unless physical examination or testing is required), thus reducing the chances of chemical trigger exposures from the staff or clinic, and enhancing overall care [54].

According to Altman and Bernstein [55], a person’s disability is not only reflected in their physical or cognitive functional limitation but also by the restriction in social participation due to a lack of accommodation [4]. In our study, the majority of people experiencing MCS report not being accommodated for their disability. People experiencing MCS often experience other’s unwillingness to adjust in the social context because of the disbelief, incomprehension, and lack of understanding of MCS. Given that people experiencing MCS already experience isolation, an accommodating environment would help mitigate the added isolation imposed by the pandemic. Accommodation requests in this study consisted of asking for scent-free policies, the use and supply of scent-free products, and using the least-toxic cleaning products, all of which are policies enforced by the Canadian Human Rights Act since 2007 for those with MCS [56]. It is interesting to note that the number of accommodation requests went down during the pandemic, and we suggest 2 possible reasons for the decline. First, during this extremely difficult time, participants may have suppressed their needs because the use of disinfectants was critical to prevent viral transmission and asking for accommodation may have resulted in further disbelief and stigmatization. Second, participants were already limiting their contact with others outside of their homes as a routine part of life and thus required less accommodation during the pandemic.

#### Social Interactions

##### Isolation

The implementation of social distancing strategies worldwide that were essential to limit the spread of the SARS-CoV-2 virus also led to an increase in social isolation for people experiencing MCS. The negative consequences of social isolation on both physical and mental health are well established, with strong associations reported between social isolation and all-cause mortality, cardiovascular illness, anxiety, and depression [56]. People experiencing MCS in our study experienced decreases in all in-person meetings with family, friends, and the community, while enduring increased social isolation consistent with reported isolation almost globally from the COVID-19 pandemic [57]. However, there was an increased level of understanding from family and a decrease in stigma from wearing personal protective equipment such as masks and gloves. Brewer and Stratton [58] suggest there may be positive outcomes of COVID-19 health measures for people living with medically unexplained conditions, in that the pandemic restrictions normalized their isolated lifestyle as one that was not only acceptable but encouraged. For example, in the workplace, many of those who were unable to work on-site due to their medical condition could work from home. Overall and importantly, lockdown and isolation may have provided insight for others, including health care professionals, into the lived experience of people experiencing MCS—one of being isolated, reticent to approach people and crowded public spaces, and wearing protective equipment as a part of daily life. Altogether, our findings on social support highlight both negative and positive outcomes of COVID-19 social distancing measures on people experiencing MCS.

##### Finding Help

One problematic outcome of COVID-19 is the increased level of difficulty in finding available human resources for support with tasks, a general trend that stemmed from workers’ fear of contracting COVID-19 [59]. In our study, human resources that were significantly more difficult to find included drivers for medical appointments, workers for home maintenance, and cleaning staff, all of whom would need to be scent free. This lack of mobility and access to others may have increased the sense of isolation of participants.

### Limitations

Our study has several limitations. We do not know if participants lived in urban or rural areas and, as such, cannot geographically contextualize the air quality findings. Also, a retrospective self-reported questionnaire is subject to recall and response bias. For example, since the period before the beginning of the COVID-19 pandemic is temporally longer than the “postpandemic” period, we cannot know how participants estimated their average when reporting “more” or “less” to any given question. Finally, given the cross-sectional nature of the study, no causal mechanism can be inferred. Future work should expand on these findings to include measures of indoor air quality alongside qualitative investigations regarding quality of life, especially in relation to the built environment.

### Conclusions and Implications

People experiencing MCS experienced significant changes to their daily life with the arrival of the COVID-19 pandemic. This study highlights unique challenges and possible benefits associated with indoor air quality, access to medical care, and social networking. Our study has important public health implications since, to our knowledge, it is the first study examining the impact of public health measures to counter the COVID-19 pandemic on the daily lives of people experiencing MCS. This study highlights a new context that people experiencing MCS must navigate in terms of their sensitivities to antimicrobial products and supports an emerging body of literature describing the risks associated with the overexposure or misuse of products used to control the COVID-19 pandemic [40,60]. Moving forward, it would be crucial to increase awareness of the chemicals contained in disinfectants and their health effects by clearly labeling the full list of ingredients and using only effective yet least toxic and fragrance-free products. We hope that our findings can guide decision makers to improve policies on accessibility, through appropriate accommodation measures, and better serve this vulnerable population in the current and future health crises. Since people experiencing MCS are particularly vulnerable in the context of the pandemic, identifying and removing barriers to access to health care should be prioritized for this population.

## Appendix

Multimedia Appendix 1 Questionnaire, response options, and scoring.

## Abbreviations

ASEQ-EHAQ: Association pour la santé environnementale du Québec—Environmental Health Association of Québec
CDC: Centers for Disease Control and Prevention
MCS: multiple chemical sensitivity
WHO: World Health Organization

## Data Availability

The data sets generated and analyzed during this study are available from the corresponding author upon reasonable request.
