# Supplementary material for: The Impact of COVID-19 Health Measures on Adults With Multiple Chemical Sensitivity: Cross-Sectional Study
Source: JMIR Form Res. 2024 Jul 17;8:e48434. doi: 10.2196/48434 (PMC11292150; doi:10.2196/48434)
Supplement: Multimedia Appendix 1 [file formative_v8i1e48434_app1.doc]

| **Item**  *Bullet points indicate that the question was answered multiple times, once for each individual factor | **Responses possible**  *Split columns indicate that the question was answered twice, for the pre-March 2020 period and post-March 2020 period | |
| --- | --- | --- |
| ***General Information – Screening Questions*** | | |
| What is your age? | Less than 18 [end of survey if selected]  18-19  13 categories of 10-year intervals from age 20 to age 79  80+ | |
| Country of residence | Canada  Other [end of survey if selected] | |
| Had you experienced MCS since at least one year prior to March 2020? | Yes  No [end of survey if selected] | |
| ***General Information – Demographics*** | | |
| What is your sex? | Female  Male  Other  Prefer not to answer | |
| What is the highest level of education you have completed? | Less than secondary school graduation  Secondary school graduation  Post-secondary education (CÉGEP, College, trade school)  Undergraduate degree (Bachelor’s)  Post-graduate certificate  Master’s degree  Doctorate or Postdoctoral degree  Prefer not to answer | |
| What is your annual household income? | < $10 000  7 categories of $10 000 intervals from $10 000 to $80 000  > $80 000  Prefer not to answer | |
| What was/is your work status? Please select the choices from the dropdown list: | Before 11/03/2020:  Employed  Unemployed  Self-employed  Student  Retired  Prefer not to answer | Currently:  Employed  Unemployed  Self-employed  Student  Retired  Prefer not to answer |
| ***General Information – MCS Health Condition*** | | |
| In which year did you start experiencing symptoms of MCS? | Categories of 1-year intervals from 2019 to 1951  1950 or before | |
| In which year did you receive a diagnosis of MCS? Please answer to the best of your recollection. | Categories of 1-year-intervals from 2020 to 1980  Before 1980 | |
| ***Physical Environment – Air Pollution*** | | |
| Were you or are you constantly or frequently exposed to the following sources of air pollution?   - Farming - Industry, powerplants - Highway or busy road - Residential or commercial smoke (wood burning, BBQs, etc.) | Before 11/03/2020:  Yes  No  Unsure | Currently:  Yes  No  Unsure |
| ***Physical Environment – Environmental Exposures & Symptoms*** | | |
| Were you or are you exposed to the following odours that enter your living environment from external sources?   - Cleaning products - Cooking odours - Disinfectants/sanitizers - Scents - Home renovation - Incense - Laundry products - Second/Third-hand Tobacco Smoke - Second/Third-hand Marijuana Smoke | Before 11/03/2020:  Yes  No  Not applicable | Currently:  Yes  No  Not applicable |
| ***Healthcare – Access to Healthcare*** | | |
| Did you have or currently have access to a family doctor? (If you have more than one doctor, please answer the questions below with reference only to your family doctor) | Before 11/03/2020:  Yes  No | Currently: Yes  No |
| Did you have or currently have access to a family doctor to address your MCS health condition? (If you have more than one doctor, please answer the questions below with reference only to your family doctor) | Before 11/03/2020:  Yes  No | Currently: Yes  No |
| List any barrier(s), if any, that you have faced or currently face in accessing a healthcare provider  (This question refers to any other healthcare professional (naturopath, osteopath, physiotherapist, etc.) and not just your family doctor, other physician(s) or dentist). Select all that apply. | Before 11/03/2020:  Air pollution exposure  Cleaning products used in the building or premises  Distance to travel  Exposures to chemicals in new masks  Exposures to unscented disinfectants or sanitizers  Outbreak of flu and /or colds  Physical distancing or wearing of masks or gloves inadequate  Exposures to scented disinfectants or sanitizers  Exposures to scents in the building or premises  Exposures to scents worn by health care workers or support staff  Mould or water damage exposure in the building or premises  Recent construction or renovations  Second/third-hand smoke exposures | Air pollution exposure  Cleaning products used in the building or premises  Distance to travel  Exposures to chemicals in new masks  Exposures to unscented disinfectants or sanitizers  Outbreak of flu and /or colds  Physical distancing or wearing of masks or gloves inadequate  Exposures to scented disinfectants or sanitizers  Exposures to scents in the building or premises  Exposures to scents worn by health care workers or support staff  Mould or water damage exposure in the building or premises  Recent construction or renovations  Second/third-hand smoke exposures |
| ***Healthcare – Satisfaction with Care*** | | |
| What is your general level of satisfaction with regards to attending a virtual or telephone meeting with your family doctor or other physician(s), to address your health issues including MCS?  Coding: Very dissatisfied =0 to Very satisfied =4 | Before 11/03/2020:  Very dissatisfied  Dissatisfied  Neutral  Satisfied  Very satisfied  Not applicable, no family doctor or other physicians | Currently:  Very dissatisfied  Dissatisfied  Neutral  Satisfied  Very satisfied  Not applicable, no family doctor or other physicians |
| What is your general level of satisfaction with regards to attending an in-person meeting with your family doctor or other physician(s), to address your health issues including MCS?  Coding: Very dissatisfied =0 to Very satisfied =4 | Before 11/03/2020:  Very dissatisfied  Dissatisfied  Neutral  Satisfied  Very satisfied  Not applicable, no family doctor or other physicians | Currently:  Very dissatisfied  Dissatisfied  Neutral  Satisfied  Very satisfied  No applicable, no family doctor or other physicians |
| ***Social Isolation*** | | |
| Have you experienced or do you experience the following? Please click on the arrow on the right to view symptoms:   - Feeling socially isolated - Participation in the local community - Stigma wearing masks, gloves or other protective equipment - Support and understanding from family - Support and understanding from friends - In-person meeting with friends - In-person meeting with family - Virtual relationships or meetings   Coding: Never =0 to Always =4 | Before 11/03/2020:  Never  Rarely  Sometimes  Often  Always | Currently:  Never  Rarely  Sometimes  Often  Always |
| What is your level of difficulty in finding scent-free workers to help you with the following tasks?   - Shopping for groceries - Driving you to appointments or for shopping - Home repairs/maintenance - Home support - House cleaning | Before 11/03/2020:  Difficult  Moderate  Easy  Not applicable | Currently:  Difficult  Moderate  Easy  Not applicable |
| ***Accommodation Measures*** | | |
| Have you requested accommodation(s) for your disability? (eg, scent-free products, least toxic disinfection, etc?) | Before 11/03/2020:  Yes  No | Currently:  Yes  No |
| Identify the reason/s for requesting accommodation(s) for your disability. Please click on the arrow on the right to view options and select all that apply: | Before 11/03/2020:  Use of scents (perfumes/  fragrances/essential oils)  Use of scented laundry products which enter your living space  Fumes from cleaning products used in common areas | Currently:  Use of scents (perfumes/  fragrances/essential oils)  Use of scented laundry products which enter your living space  Fumes from cleaning products used in common areas |
| Identify your request(s) for accommodation(s) for your disability. Please click on the arrow on the right to view options: | Before 11/03/2020:  Scent-free policies  Use of scent-free, least-toxic cleaning products  Supply of scent-free, least-toxic soaps, disinfectants or sanitizers | Currently:  Scent-free policies  Use of scent-free, least-toxic cleaning products  Supply of scent-free, least-toxic soaps, disinfectants or sanitizers |
| Were you reasonably accommodated following your request(s)? | Before 11/03/2020:  Yes  No | March 11, 2020 to present:  Yes  No |
